# Vesical Calculus in a Urinary Typhoid Carrier

**Published:** 1920-09

**Authors:** Duncan Wood

**Affiliations:** Visiting Surgeon, Bath Pensions Hospital; Surgical Registrar, Bristol General Hospital


### VESICAL CALCULUS IN A URINARY TYPHOID CARRIER.

BY

DUNCAN WOOD, F.R.C.S.,

*Visiting Surgeon, Bath Pensions Hospital; Surgical Registrar, Bristol General Hospital.*

---

THE bacterial causation of urinary calculi is well recognised in the so-called secondary stones such as the triple phosphates. In these cases the bacteria split up the urea, turning the urine alkaline and ammoniacal, and precipitating crystals of triple phosphates and of calcium carbonate.

The etiology of the primary calculi of calcium oxalate and uric acid is not so clear. Their bacterial origin has been suggested, but very few cases have been published which throw light on the subject. The following is recorded with a view to increasing our knowledge on this subject.

A.B., a male, age 25, was working as a schoolmaster before the War. In November, 1914, he was inoculated twice with typhoid vaccine with an interval of ten days between the two injections. In November, 1915, he was taken ill at Lemnos, with diarrhœa and passing blood in the stools. He also had marked frequency of micturition, to such a degree that if he stood up he had incontinence of urine. He did not notice any blood in his urine at this time.

In December, 1915, he was labelled "nephritis" and invalided to England. On arrival he was found to have typhoid bacilli in his urine. He was treated with dieting and urotropine. Between January and March, 1916, he received eight injections of typhoid vaccine. Between March and August, 1916, sixteen attempts to find the typhoid bacilli in the fæces were all negative. In May, 1918, typhoid bacilli were still present in the urine. During the following eight weeks he had six inoculations with an autogenous paratyphoid A. vaccine. He was also given urotropine gr. v. three times a day by the mouth. At the end of this course typhoid bacilli were still present in the urine. In the summer of 1918 he first noticed blood in the urine. The hæmaturia appeared in attacks lasting a few days at a time.

In January, 1920, the hæmaturia became continuous; the blood was bright in colour and appeared at the end of micturition. Pain was present during micturition, worse at the end of the act and referred to the end of the penis. At no time was there pain in the loin, either dull or sharp in character. The frequency was every two hours during the day and twice at night. In February, 1920, a stone was felt with a sound. The X-ray showed a stone in the bladder, none in either kidney or ureter. The urine was sp. gr. 1020, acid, containing a trace of albumen and, microscopically, a few red-blood corpuscles and leucocytes. B. typhosus was grown from a catheter specimen of the urine, but the fæces were negative. There was no fever. Cystoscopy showed no changes in the bladder wall.

February 14th, 1920, suprapubic cystotomy was performed and the stone removed. The incision in the bladder wall was closed by two layers of catgut, and a rubber glove drain inserted in the prevesical space. Slight leakage of urine occurred on the fifth day, but the wound rapidly healed. The stone was 2 cm. by 1.5 cm. in size, mulberry shaped, and chemically consisted of calcium oxalate. It was placed in 1 in 20 carbolic acid for one hour, was then washed in sterile saline, cut across aseptically, and a culture taken from scrapings from the centre of the stone. The culture grew b. coli. Treatment with urotropine gr. x. three times a day was continued.

On February 27th, 1920, B. typhosus was still present in the urine. On March 19th, 1920, a culture was negative. On April 10th, 1920, the culture was positive. On May 2nd, 1920, culture taken from urine from right kidney was negative. Agglutination of blood for B. typhosus 1 in 50 ; for B. paratyphosus A. 1 in 1000 ; for B. paratyphosus B. negative. On July 1st, 1920, treatment with urotropine was stopped. On August 3rd, 1920, a culture of urine from the bladder was negative, also from the left kidney.

It appears from the original diagnosis of the disease that the onset was associated with an acute nephritis. No casts have been found in the urine during this year. A febrile albuminuria is commoner, and this would allow the bacilli to pass through the kidneys. Osler states that typhoid bacilluria occurs in about 30 per cent. of all cases in the acute stage. Judging from the agglutination results, the infecting organism in this case was the B. paratyphosus A. Webb Johnson found that B. paratyphosus B. was more frequently the cause of cystitis than the other organisms of the group. In this case the infection has not been localised to either kidney. Webb Johnson points out that even if it can be localised to one kidney, it may merely be that it is more permeable to typhoid bacilli than the other kidney. He suggests that the foci which harbour the disease are the bone marrow and the spleen.

Was the stone formed in either kidney ? It is generally considered that calcium oxalate calculi are renal in origin, but this patient has never had pain in his loins, nor renal colic. The difficulties surrounding the final cure of these patients have been pointed out by Davies and Walker Hall, in whose case the patient was free from bacilluria for three months, and then recurrence occurred.

In conclusion, my thanks are due to Dr. Waterhouse for the bacteriology in this paper.

### REFERENCES.

Osler, *Practice of Medicine*, p. 88, 6th edition.
Webb Johnson, *Lancet*, 1917, ii. 819.
Walker Hall, *Lancet*, 1913, ii. 1,307.

